# Calcitriol inhibits osteoclastogenesis in an inflammatory environment by changing the proportion and function of T helper cell subsets (Th2/Th17)

**DOI:** 10.1111/cpr.12827

**Published:** 2020-05-13

**Authors:** Chun‐Sheng Bi, Xuan Li, Hong‐Lei Qu, Li‐Juan Sun, Ying An, Yong‐Long Hong, Bei‐Min Tian, Fa‐Ming Chen

**Affiliations:** ^1^ State Key Laboratory of Military Stomatology and National Clinical Research Center for Oral Diseases Department of Periodontology School of Stomatology Fourth Military Medical University Xi'an China; ^2^ Department of Periodontics Stomatology Hospital Zhejiang University School of Medicine Hangzhou China; ^3^ Stomatology Center Shenzhen Hospital of Southern Medical University Shenzhen China

**Keywords:** calcitriol, dendritic cells, immunomodulation, osteoclastogenesis, T helper cells

## Abstract

**Objectives:**

Previously, we found that by regulating T helper (Th) cell polarization, calcitriol intervention inhibited lipopolysaccharide (LPS)‐induced alveolar bone loss in an animal periodontitis model, but the underlying cellular events remain unknown.

**Materials and methods:**

In this study, mouse Th cells were incubated in an inflammatory environment in the presence of dendritic cells (DCs) and LPS. Then, the potential of the Th cells to undergo Th2/Th17 polarization, the RANKL expression of the polarized Th cells and the subsequent influences of the polarized Th cells on RAW264.7 cell osteoclastogenesis in response to calcitriol administration were assessed. Finally, the effects of calcitriol on antigen presentation by DCs during these cellular events were evaluated.

**Results:**

In response to calcitriol administration, Th cells in an inflammatory environment exhibited an enhanced potential for Th2 polarization along with a decreased potential for Th17 polarization. In addition, RANKL expression in Th17‐polarized cells was largely inhibited. Furthermore, inflammation‐induced osteoclastogenesis in RAW264.7 cells was suppressed following coculture with calcitriol‐treated Th cells. During these cellular events, increased expression of Th2 promoters (such as OX‐40L and CCL17) and decreased expression of Th17 promoters (such as IL‐23 and IL‐6) were found in DCs.

**Conclusions:**

Calcitriol can inhibit osteoclastogenesis in an inflammatory environment by changing the proportion and function of Th cell subsets. Our findings suggest that calcitriol may be an effective therapeutic agent for treating periodontitis.

## 1 INTRODUCTION

Chronic periodontitis is characterized by local inflammation‐induced bone loss due to the interaction between the host defence mechanism and plaque bacteria on the supporting tissues of the teeth.^1^, ^2^ During this inflammatory process, the host inflammatory response inevitably exerts an impact that induces systematic skeletal changes in alveolar bone. Specifically, the interplay between the immune system network and the receptor activator of nuclear factor‐κB ligand (RANKL)/receptor activator of nuclear factor‐κB (RANK)/osteoprotegerin (OPG) pathway has been recognized to play essential roles during the progressive damage of the bone until tooth loss.^1^

In terms of the immune system network, T helper (Th) cells, the subsets of which are determined by immunity‐associated antigen‐presenting cells such as dendritic cells (DCs), can play both damaging and protective roles in bone loss by regulating the RANKL‐OPG system.^3^, ^4^ Classically, Th cells can be divided into four types (Th1, Th2, Th17 and regulatory T cell (Treg)). Among these types, Th17 subsets with proinflammatory and pro‐osteoclastogenic characteristics are reported to enhance leucocyte influx into periodontal tissues and intensify bone lesions through osteoclast precursor maturation and enhanced local RANKL levels.^5^, ^6^, ^7^ In contrast, the Th2 phenotype and its characteristic cytokine, IL‐4, can attenuate periodontal tissue destruction by inhibiting proinflammatory cytokine and RANKL expression.^7^, ^8^, ^9^, ^10^, ^11^ Moreover, an increasing number of studies have also found that the functions of Th cells are closely related to RANKL‐induced osteoclast differentiation.^8^, ^12^, ^13^ In this context, researchers have attempted to attenuate inflammation‐induced tissue destruction by modulating Th cells towards anti‐inflammatory polarization. For example, promoting Th2 polarization and attenuating Th17 polarization by using bioactive immunomodulator agents, such as calcitriol, may be a practical strategy to inhibit tissue destruction.^14^, ^15^, ^16^

Calcitriol has been confirmed to be an essential regulator in bone mineralization that functions by modulating the calcium and phosphate levels in the systemic circulation.^17^, ^18^ A deficiency in calcitriol can exacerbate osteopaenia and increase the risk of fracture in osteoporosis.^18^, ^19^ In addition to its widely known effects on bone homeostasis, calcitriol has also recently been shown to reverse the development of disease by modulating immune functions.^20^ For example, calcitriol can exploit the antigen‐presenting ability of DCs to regulate the polarization states of T cells, which contributes to inflammation resolution and prevents transplant rejection.^21^, ^22^ In our previously published study, we found that calcitriol supplementation could suppress periodontitis‐induced bone destruction by promoting Th2 polarization and inhibiting Th17 polarization in an animal periodontitis model.^14^ However, how calcitriol‐induced Th cell polarization is associated with the activation of osteoclasts still requires further investigation. Therefore, the aim of the present study was to analyse the effects of calcitriol intervention on Th cell polarization and RANKL expression in an inflammatory environment in the presence of DCs and lipopolysaccharide (LPS). Then, the effect of Th cell polarization on RAW264.7 cell osteoclastogenesis was evaluated in vitro. To investigate whether calcitriol also influences the cellular behaviour of DCs, the biological effects of calcitriol on antigen presentation by DCs were assessed. Overall, our research was designed to uncover the underlying mechanism by which calcitriol affects LPS‐induced osteoclastogenesis in vitro.

## 2 MATERIALS AND METHODS

### 2.1 Isolation and purification of mouse Th cells and DCs

In the present study, both Th cells and DCs were obtained from freshly harvested spleens from C57BL/6 mice (aged 6‐8 weeks) that were purchased from the Laboratory Animal Research Centre of the Fourth Military Medical University (FMMU; Xi'an, China). The use of mice and all experimental procedures were approved by the Intramural Animal Use and Care Committee of the FMMU (protocol number: 2016016). Briefly, after mice were euthanized, freshly isolated spleens were ground in cold phosphate‐buffered saline (PBS; HyClone) and filtered through strainers to obtain a single‐cell suspension. Then, the cells were resuspended and incubated in complete RPMI 1640 medium (Thermo Fisher) containing 10% foetal bovine serum (FBS; Sijiqing), 1% penicillin (Invitrogen, Life Technologies) and 1% streptomycin (Invitrogen). After a culture period of 6 hours, the obtained cells were processed to isolate CD4+ Th cells according to the specifications of the Easy Sep™ Mouse CD4+ T Cell Isolation Kit (19852, STEMCELL Technologies Inc). For isolating and purifying DCs, the resuspended splenocytes were cultured in complete RPMI 1640 medium for 12 hours. After the removal of non‐adherent cells in the supernatant, the adherent cells were incubated in complete medium containing 500 ng/mL GM‐CSF (CK02; Novoprotein Scientific Inc) and 200 ng/mL IL‐4 (CK15; Novoprotein Scientific Inc). Following a culture period of 3 days, DCs could be successfully isolated using the EasySep™ Mouse CD11c Positive Selection KitⅡ (18780, STEMCELL Technologies Inc). The purity of isolated Th cells and DCs was detected with a flow cytometry assay by using anti‐CD4 PE‐conjugated (100408; BioLegend) and anti‐CD11c PE‐conjugated (117307; BioLegend) antibodies, respectively.^16^, ^23^ Finally, the purified cells were cultured at 37°C in 95% humidity and 5% CO_2_, with medium changes performed every 2 days.

### 2.2 Determination of the concentration of calcitriol used in cell cultures

First, a physiological saline solution was used to dissolve calcitriol (Roche Pharmaceuticals Ltd.). Then, we screened an appropriate concentration of calcitriol according to CCK8 assays. Briefly, both DCs and Th cells were seeded in triplicate in a 96‐well plate at a density of 2 × 10^3^ cells/well. After cell adhesion occurred, the cells were incubated with various concentrations of calcitriol (0, 1, 10 or 100 nmol/L). The CCK‐8 assay was then performed on days 1, 2, 3, 4 and 5. For each test, 10 μL of CCK‐8 solution was added to each test well, followed by an incubation at 37°C for 3 hours.^24^ The optical absorbance (OD) value of the test well was then measured at 450 nm using a microplate reader (Infinite M200 Pro).

### 2.3 Study design

Lipopolysaccharide, as a virulence factor from gram‐negative bacteria, was used as a pathogenic agent to induce animal periodontitis in our previous research and other reports.^12^, ^14^ In this study, DCs in complete RPMI 1640 medium supplemented with 1 μg/mL LPS (from *Porphyromonas gingivalis*, SMB00610, Sigma) were designated as the inflammatory environment. Th cells (2 × 10^5^ cells) and DCs (1 × 10^6^ cells) from the same mouse were placed in the upper chamber and lower chamber of a transwell system, respectively, with 1 μg/mL LPS (LPS + DC group). The appropriate dose of calcitriol was applied in the coculture transwell system (LPS + DC + Cal group). Th cells incubated in complete RPMI 1640 medium supplemented with 1 μg/mL LPS (without coculturing with DCs or adding calcitriol) were set up as the control (LPS group).

### 2.4 Effect of calcitriol administration on Th cell proliferation in an inflammatory environment

To investigate the proliferation of Th2 cells (IL‐4 labelled) and Th17 cells (IL‐17 labelled), 5‐carboxyfluorescein diacetate succinimidyl ester (CFSE)‐based flow cytometry was performed. Briefly, 5 μmol/L CFSE working solution (21888, Sigma) was added to each well. After a 5‐day incubation, cells were fixed with Intracellular Fixation & Permeabilization Buffer (88‐8823‐88; eBioscience) and then immunostained using an anti‐IL‐4 (APC, 355005; BioLegend) or anti‐IL‐17 (APC, 146307; BioLegend) antibody to identify Th2 and Th17 cells, respectively. Finally, the proliferation of the CFSE pre‐stained IL‐4 or IL‐17 cells was analysed with a Beckman Coulter Epics XL flow cytometer at FITC. In addition, to explore whether the antigen‐presenting ability of DCs was changed in both the direct and indirect co‐incubation systems, CFSE‐based flow cytometry was performed to detect the proliferation of Th2 and Th17 cells in the three groups (LPS group, LPS + DC group, LPS + DC + Cal group) without the transwell co‐incubation system.

### 2.5 Effect of calcitriol administration on Th cell polarization in an inflammatory environment

The Th cells in the three groups (LPS group, LPS + DC group, LPS + DC + Cal group) were subjected to a 5‐day incubation, and the polarization states of these Th cells were evaluated by flow cytometry.^8^, ^25^, ^26^ Briefly, after immunostaining with an anti‐CD4 PE‐conjugated antibody, Th cells were fixed with Intracellular Fixation & Permeabilization Buffer and then immunostained using an anti‐IL‐4 or anti‐IL‐17antibody to identify Th2 and Th17 cells, respectively. Finally, the immunostained Th cells were detected and analysed with a Beckman Coulter Epics XL flow cytometer. In addition, the expression of Th2 polarization‐related genes and proteins (GATA3, STAT5 and IL‐4)^27^, ^28^ and Th17 polarization‐related genes and proteins (STAT3, RORγT and IL‐17)^16^, ^29^, ^30^, ^31^ in the cultured Th cells was determined by quantitative real‐time polymerase chain reaction (qRT‐PCR) and Western blot analysis (for details, see Sections 2.8 and 2.9).

### 2.6 Effect of calcitriol administration on RANKL expression in Th cells in an inflammatory environment

In parallel to the investigation of cell polarization, the gene and protein levels of RANKL in the Th cells (LPS group, LPS + DC group and LPS + DC + Cal group) following a 5‐day incubation were determined by qRT‐PCR and Western blot analysis (for details, see Sections 2.8 and 2.9). In addition, immunofluorescence and flow cytometry assays were performed to assess the proportion of IL‐17^+^/RANKL^+^ cells.^13^ Briefly, after Th cell smears were prepared, Th cells were incubated with primary antibodies against RANKL (ab45039; Abcam) and IL‐17 (ab180904; Abcam) overnight at 4°C and then incubated with secondary antibodies (Alexa Fluor 488‐conjugated goat anti‐rabbit and Alexa Fluor 647‐conjugated goat anti‐mouse) for 50 minutes in the dark. Finally, the cells were counterstained with 4′,6‐diamidino‐2‐phenylindole (DAPI) and then visualized and imaged with a fluorescence microscope (NIKON ECLIPSE C1, Nikon DS‐U3). Similarly, for detecting the proportion of IL‐17^+^/RANKL^+^ cells by flow cytometry, cells were immunostained with PE‐conjugated anti‐mouse RANKL (IK22/5, BioLegend) and APC‐conjugated anti‐mouse IL‐17 antibodies, and the immunostained IL‐17^+^/RANKL^+^ cells were detected and analysed with a Beckman Coulter Epics XL flow cytometer (Beckman Coulter).^32^

### 2.7 The effect of calcitriol‐treated Th cells on RAW264.7 cell osteoclastogenesis

The mouse preosteoclast cell line RAW264.7, purchased from the Chinese Academy of Sciences (Shanghai, China), was cultured in complete RPMI 1640 medium. The medium was changed every other day. To explore the effect of calcitriol‐treated Th cells on the osteoclastogenesis of osteoclasts, Th cells from different groups (LPS group, LPS + DC group and LPS + DC + Cal group) were cocultured with RAW246.7 cells in a transwell system (Th cells were plated in the upper chamber, and RAW246.7 cells were plated in the lower chamber; the number of cells in each chamber was 2 × 10^4^). Following a 5‐day incubation, the osteoclast activity of RAW264.7 cells was detected by TRAP staining.^32^ For TRAP staining, cell slides were fixed with 4% paraformaldehyde for 15 minutes and then stained at 37°C for 90 minutes according to the manufacturer's protocol for a TRAP kit (G1050; Servicebio). Finally, light microscopy was applied to image and count TRAP‐positive multinucleated cells. Furthermore, the levels of osteoclastogenesis‐related genes and proteins (such as NFATc1 and MMP‐9) were determined by qRT‐PCR and Western blot analysis (for details, see Sections 2.8 and 2.9).^33^

### 2.8 The effects of calcitriol administration on the antigen‐presenting function of DCs

The antigen‐presenting activity of DCs related to Th cell polarization was analysed using flow cytometry.^34^ First, DCs were incubated in complete RPMI 1640 medium supplemented with 1 μg/mL LPS in the absence (LPS group) or presence (LPS + Cal group) of 10 nmol/L calcitriol, while DCs cultured in only complete RPMI 1640 medium were designated the control (control group). Following a 5‐day incubation, the cells were harvested and incubated at 4°C for 1 hour with the following primary antibodies: mouse anti‐MHCⅡ (FITC, 109905; BioLegend) and anti‐CD86 (APC, 105011; BioLegend). Finally, the proportion of MHCⅡ^+^/CD86^+^ cells was detected and analysed with a Beckman Coulter Epics XL flow cytometer.

### 2.9 The effects of calcitriol administration on the expression of Th2 or Th17 promoters in DCs

To clarify the role of DCs in calcitriol‐mediated Th cell polarization, DCs were incubated in various conditions (in parallel with the incubation of Th cells, a control group, an LPS group and an LPS + Cal group were designed for DC incubation). After the Th cell polarization‐related antigen‐presenting function of DCs was determined by flow cytometry, qRT‐PCR and Western blot analyses were used to further detect the expression levels of Th2 promoters (such as OX‐40L and CCL17) or Th17 promoters (such as IL‐23 and IL‐6) in the DCs^35^, ^36^ (for details, see Sections 2.8 and 2.9).

### 2.10 qRT‐PCR

To determine the expression levels of related genes in cells, total RNA was extracted from cells using a TRIzol solution according to the manufacturer's instructions (Invitrogen). A reverse transcription reaction for cDNA synthesis was performed using PrimeScript™ RT Master Mix (Perfect Real Time; Takara). After the cDNA samples were diluted 5‐fold, qRT‐PCR was carried out by using SYBR Green Master Mix (Roche) and the CFX Connect™ Real‐Time PCR Detection System (Bio‐Rad). Amplification was performed in a 10 μL PCR system containing 2 μL of cDNA, 2.2 μL of ddH_2_O, 5 μL of SYBR mix, 0.4 μL of reverse primer and 0.4 μL of forward primer. GAPDH was used as the housekeeping gene for normalization. The primer sequences used in this study are shown in Table 1.

**TABLE 1:** Primer sequences used in the present study

GENE	Forward (5′‐3′)	Reverse (5′‐3′)
*GATA3*	GAGGTGGACGTACTTTTTAACATCG	GGCATACCTGGCTCCCGT
*STAT5*	CAGAGTCGGTGACGGAGGAGAA	GCACGGTGGCAGTAGCATTGT
*IL‐4*	ACCACAGAGAGTGAGCTCGT	AGGCATCGAAAAGCCCGAAA
*RORγT*	CCGCTGAGAGGGCTTCAC	TGCAGGAGTAGGCCACATTACA
*STAT3*	GAGGTGGACGTACTTTTTAACATCG	GGCATACCTGGCTCCCGT
*IL‐17*	AACACTGAGGCCAAGGACTTC	GTCTTCATTGCGGTGGAGAGT
*RANKL*	CGCTCGTGTTTCTGGACATC	GGGGCTGCAGTATAGACACT
*NFATc1*	TCTGGGAGATGGAAGCAAAGACTG	AGGGCTATCACGTGGTG TGAAGAG
*MMP‐9*	GATCCCCAGAGCGTCATTC	CCACCTTGTTCACCTCATTTTG
*GAPDH*	ACCACAGTCCATGCCATCAC	TCCACCACCCTGTTGCTGTA
*IL‐6*	ATGAACTCCTTCTCCACAAGC	CTACATTTGCCGAAGAGCCCTCAGGCTGGACTG
*OX‐40L*	CCTGATACTCTCTGCGAACACC	AAGAACCTGTGTCCCGTCCA
*CCL17*	AGATCTTCCACGAACACCCC	AATAGGTTGGACCTCATGGC
*IL‐23*	GTCTCGGATTCTGGAGTGACGA	TGGTCTTTCTTGGGTT

### 2.11 Western blot analysis

To determine the expression levels of related proteins in cells, total protein was extracted by using a lysis buffer (Sigma‐Aldrich) and boiled for 10 minutes. Protein samples containing the same quantity (40 μg) were subjected to Western blot analysis according to protein concentrations measured by a BCA assay (Beyotime). Subsequently, the protein samples were separated by sodium dodecyl sulphate‐polyacrylamide gel electrophoresis (SDS‐PAGE) and transferred onto polyvinylidene fluoride (PVDF) membranes. After blocking in a 5% milk solution for 2 hours, the membranes were incubated with the following primary antibodies: anti‐GATA3 (ab110093, Abcam), anti‐p‐GATA3 (SAB4301577, Sigma) anti‐STAT5 (ab215367, Abcam), anti‐p‐STAT5 (ab32364, Abcam), anti‐IL‐4 (ab11524, Abcam), anti‐RORγT (04‐959, Sigma), anti‐STAT3 (GB12176, Servicebio), anti‐IL‐17 (PRS4877, Sigma), anti‐RANKL (orb542071, Biorbyt), anti‐NFATc1 (orb381911, Biorbyt), anti‐MMP‐9 (GB12132‐1, Servicebio), anti‐IL‐6 (ab208113, Abcam), anti‐IL‐23 (orb74717, Biorbyt), anti‐OX40L (ab156285, Abcam) and anti‐CCL17 (SAB3500860, Sigma) overnight at 4°C. Appropriate anti‐rabbit or anti‐mouse horseradish peroxidase (HRP)‐conjugated secondary antibodies (Cowin Biotech Co., Ltd.) were subsequently used. Finally, the blots were developed using the Western Light Chemiluminescent Detection System (Peiqing), and quantification analysis was performed with ImageJ. The grey value of each targeted protein was normalized to that of actin before comparison (Boster; BM0627).

### 2.12 Statistical analysis

In this study, all experiments were performed in triplicate, and all values are expressed as the mean ± standard deviation (*SD*). One‐way analysis of variance (ANOVA) and Dunn's post hoc tests were applied to analyse comparisons among groups (no less than three groups). Statistical analysis was performed using SPSS software 17.0. Differences were considered statistically significant when *P* < .05.

## 3 RESULTS

### 3.1 Calcitriol facilitated the polarization of Th cells towards Th2 phenotypes while inhibiting the polarization of Th cells towards Th17 phenotypes in an inflammatory environment

**FIGURE 1:**
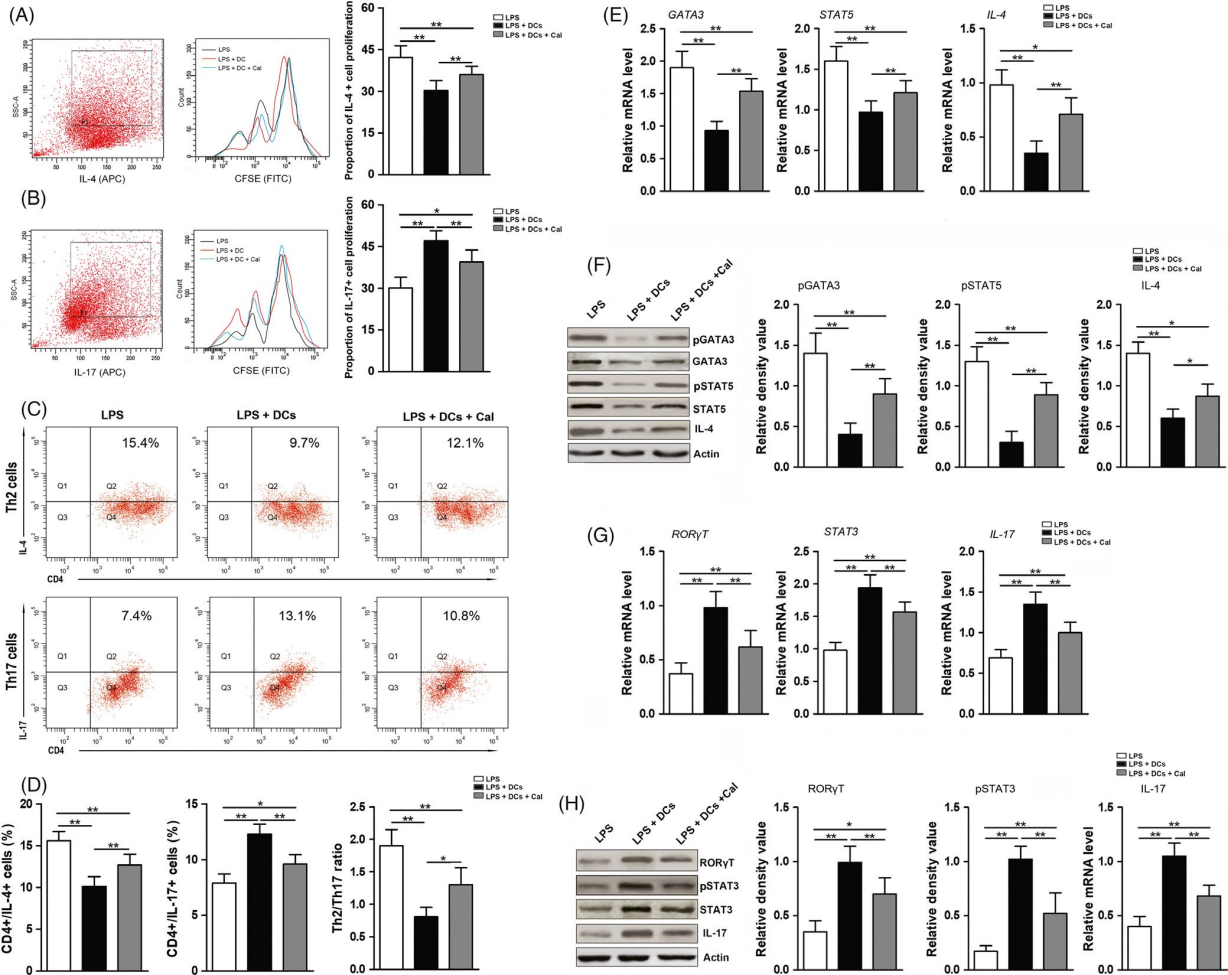
Calcitriol facilitated the polarization of Th cells towards the Th2 phenotype while inhibiting the polarization of Th cells towards the Th17 phenotype in an inflammatory environment. A, CFSE‐based flow cytometry was used to assess the proliferation of Th2 cells (IL‐4 labelled) in the indirect co‐incubation (transwell) system. B, CFSE‐based flow cytometry was used to assess the proliferation of Th17 cells (IL‐17 labelled) in the indirect co‐incubation (transwell) system. C, Representative flow cytometry plots of CD4^+^/IL‐4^+^ Th2 and CD4^+^/IL‐17^+^ Th17 cells following incubations in various conditions (LPS group, LPS + DC group and LPS + DC + Cal group). D, Quantification of the proportions of CD4^+^/IL‐4^+^ Th2 and CD4^+^/IL‐17^+^ Th17 cells (calculated by flow cytometry) and the Th2/Th17 ratio. E, mRNA levels of Th2 polarization‐related markers (*GATA3*, *STAT5* and *IL‐4*) in Th cells following incubations in various conditions (LPS group, LPS + DC group and LPS + DC + Cal group). F, Protein levels of Th2 polarization‐related markers (p‐GATA3, STAT5 and IL‐4) in Th cells (determined by Western blotting) following incubations in various conditions (left panel) and semi‐quantitative analysis of the protein expression levels (normalized to the level of actin) in terms of the relative grey density (right panel). G, The mRNA levels of Th17 polarization‐related markers (*STAT3*, *RORγT* and *IL‐17*) in Th cells following incubations in various conditions (LPS group, LPS + DC group and LPS + DC + Cal group). H, The protein levels of Th17 polarization‐related markers (p‐STAT3, RORγT and IL‐17) in Th cells (determined by Western blotting) following incubations in various conditions (left panel) and semi‐quantitative analysis of the protein expression levels (normalized to the level of actin) in terms of the relative grey density (right panel). The data are shown as the mean ± SD; *P < .05 and **P < .01 represent significant differences between the indicated columns

Flow cytometry analysis showed that the purities of CD4+ T cells and DCs isolated from mouse spleens with immunomagnetic beads were above 90% (Figure S1A). When Th cells and DCs were treated with various concentrations of calcitriol (0, 1, 10 or 100 nmol/L), a CCK‐8 assay revealed that the cell viabilities of the Th cells and DCs treated with calcitriol were reduced in a dose‐dependent manner. Our previous research and that of others^14^, ^16^ indicated that 10 nmol/L calcitriol has a strong pharmacological effect but no adverse effects on cell viability; thus, it was selected as the appropriate concentration in the following assays (Figure S1B). In this study, DCs in complete RPMI 1640 medium supplemented with LPS were designated the inflammatory environment (LPS + DC group), and the effect of 10 nmol/L calcitriol on Th cell polarization was assessed in this inflammatory environment (LPS + DC + Cal group). To evaluate the antigen‐presenting ability of DCs, CFSE staining was performed. In both co‐incubation systems (with or without transwell), the Th2 cells in the LPS + DC + Cal group were more capable of division than those in the LPS + DC group, while the Th17 cells in the LPS + DC + Cal group were less capable of division than those in the LPS + DC group (Figure 1A,B and Figure S2A,B). Moreover, in terms of flow cytometric analysis, a higher proportion of Th2 cells marked with CD4 and IL‐4 and a lower proportion of Th17 cells marked with CD4 and IL‐17 were found in the LPS + DC + Cal group compared with the LPS + DC group, and the Th2/Th17 ratio was increased by the addition of calcitriol as well (Figure 1C,D). Then, specific transcription factors and products related to Th2 and Th17 polarization were detected using qRT‐PCR and Western blot analyses. At the mRNA level, significantly higher levels of Th2‐specific transcription factors (*GATA3* and *STAT5*) and their product (*IL‐4*) were found in the LPS + DC + Cal group compared with the LPS + DC group, while much lower expression levels of Th17‐specific transcription factors (*STAT3* and *RORγT*) and their product (*IL‐17*) were found in the LPS + DC + Cal group compared with the LPS + DC group (Figure 1E,H). Similarly, the results for the Western blot analysis showed that calcitriol intervention could upregulate the levels of phosphorylated GATA3 (p‐GATA3), phosphorylated STAT5 (p‐STAT5) and IL‐4 but downregulate the levels of phosphorylated STAT3 (p‐STAT3), RORγT and IL‐17 (Figure 1F,G).

### 3.2 Calcitriol inhibited RANKL expression in Th cells in an inflammatory environment

**FIGURE 2:**
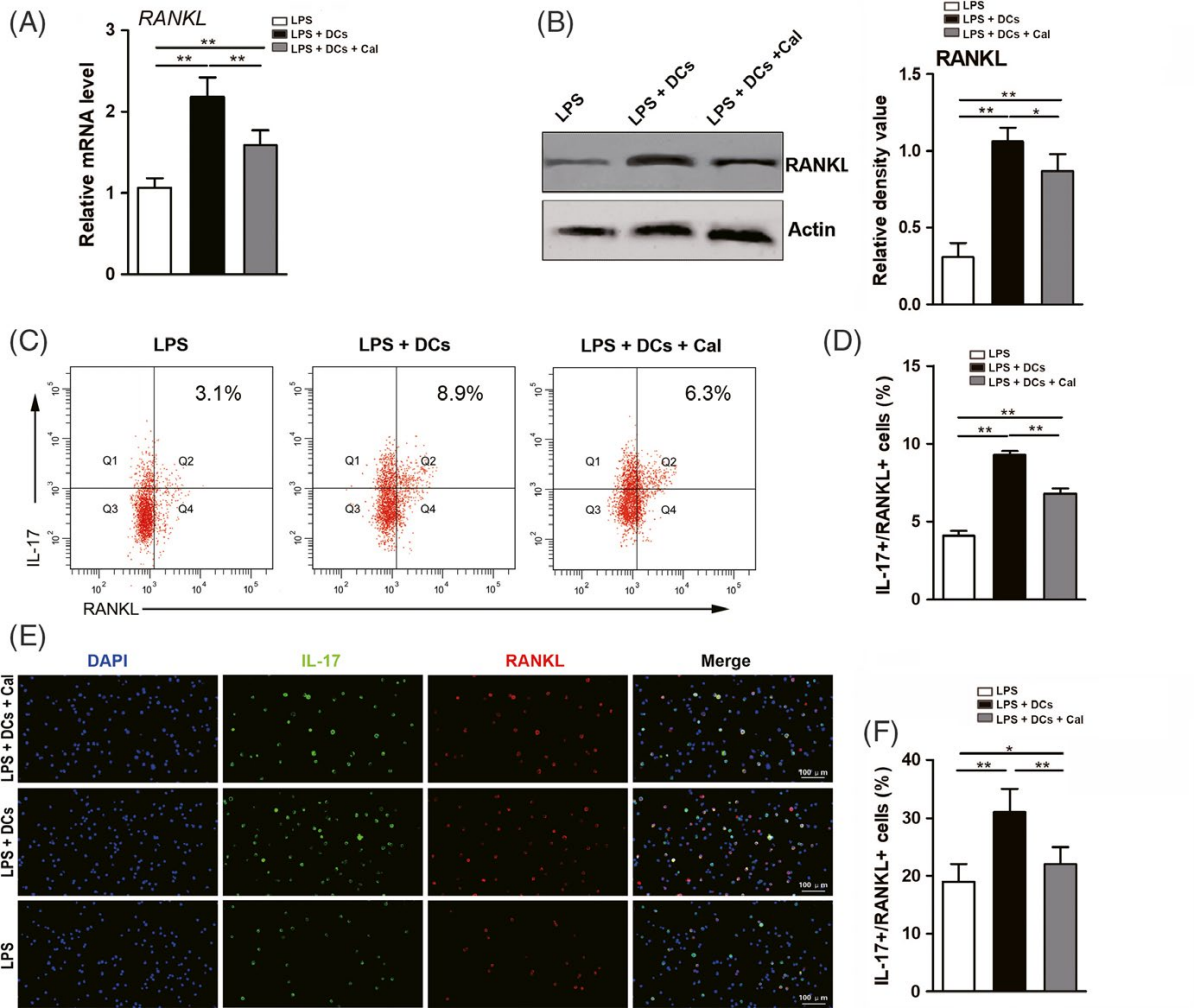
Suppressive effect of calcitriol on RANKL expression in Th cells in an inflammatory environment. A, mRNA level *of RANKL* in Th cells (determined by qRT‐PCR) following incubations in various conditions (LPS group, LPS + DC group and LPS + DC + Cal group). B, Protein level of RANKL in Th cells (determined by Western blotting) following incubations in various conditions (LPS group, LPS + DC group and LPS + DC + Cal group; left panel) and semi‐quantitative analysis of the protein expression level (normalized to the level of actin) in terms of the relative grey density (right panel). C, Representative flow cytometry plots of IL‐17^+^/RANKL^+^ Th cells following incubations in various conditions (LPS group, LPS + DC group and LPS + DC + Cal group). D, Quantification of the proportion of IL‐17^+^/RANKL^+^ Th cells (assessed by flow cytometry). E, Representative immunofluorescence images of IL‐17^+^/RANKL^+^ Th cells (cell nucleus, blue fluorescence; IL‐17 protein, green fluorescence; RANKL protein, red fluorescence; scale bar: 100 μm). F, Quantification of the proportion of IL‐17^+^/RANKL^+^ Th cells (calculated from an immunofluorescence assay). The data are shown as the mean ± SD; *P < .05 and **P < .01 represent significant differences between the indicated columns

Following analysis of the polarization of Th cells, the effect of calcitriol on RANKL expression in Th cells was assessed. In terms of qRT‐PCR and Western blot analyses, significantly higher RANKL expression levels were found in the LPS + DC group compared with the LPS group, while the addition of calcitriol downregulated the elevated RANKL expression levels in Th cells in the inflammatory environment (Figure 2A,B). Consistently, flow cytometry and immunofluorescence assays showed that the proportion of IL‐17+/RANKL+ Th cells was increased when cells were incubated in the inflammatory environment (LPS + DC group). When the cells were incubated with calcitriol, the increase in the proportion of IL‐17^+^/RANKL^+^ Th cells was abolished (Figure 2C–F).

### 3.3 Calcitriol‐treated Th cells inhibited RAW264.7 cell osteoclastogenesis

**FIGURE 3:**
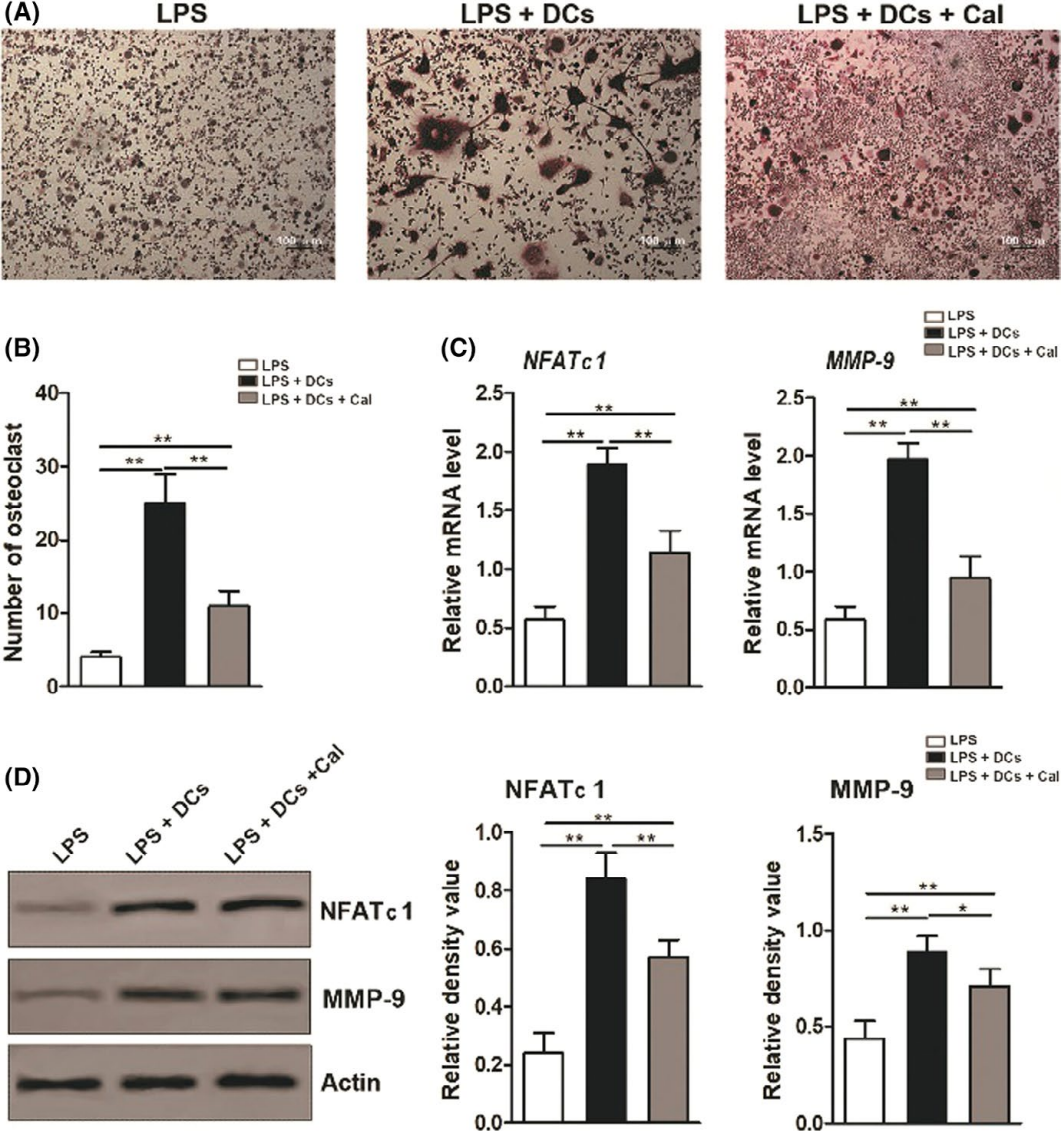
Inhibitory effects of calcitriol‐treated Th cells on RAW264.7 cell osteoclastogenesis in a transwell coculture system. A, Representative images of the TRAP staining of RAW264.7 cells following coculture with Th cells from the LPS group, LPS + DC group or LPS + DC + Cal group (multinuclear, red TRAP‐positive cells with a bifurcate prominence; scale bar: 100 μm). B, Quantitative analysis of TRAP‐positive RAW264.7 cells following coculture with Th cells from different groups (LPS group, LPS + DC group and LPS + DC + Cal group). C, The mRNA levels of osteoclastogenesis‐related markers (*NFATc1* and *MMP‐9*) in RAW264.7 cells following coculture with Th cells from the LPS group, LPS + DC group or LPS + DC + Cal group. D, Protein levels of osteoclastogenesis‐related markers (NFATc1 and MMP‐9) in RAW264.7 cells (determined by Western blotting) following coculture with Th cells from the LPS group, LPS + DC group or LPS + DC + Cal group (left panel) and semi‐quantitative analysis of the protein expression level (normalized to the level of actin) in terms of the relative grey density (right panel). The data are shown as the mean ± SD; *P < .05 and **P < .01 represent significant differences between the indicated columns

To investigate the roles of calcitriol‐treated Th cells in RAW264.7 cell osteoclastogenesis, Th cells were preincubated in various conditions (LPS group, LPS + DC group and LPS + DC + Cal group) and then cocultured with RAW264.7 cells in a transwell coculture system. In this coculture system, more TRAP‐positive RAW264.7 cells were found in the LPS + DC group compared with the LPS group. When RAW264.7 cells were cocultured with calcitriol‐treated Th cells, the increased TRAP staining of RAW264.7 cells in the inflammatory environment was largely inhibited (Figure 3A,B). Moreover, qRT‐PCR and Western blot analyses showed that coculture with Th cells in the inflammatory environment (LPS + DC group) induced increased expression of osteoclastogenesis‐related markers (such as NFATc1 and MMP‐9) in RAW264.7 cells, whereas coculture with calcitriol‐treated Th cells downregulated the increased expression levels of the osteoclastogenesis‐related markers in RAW264.7 cells (Figure 3C,D).

### 3.4 Modulatory effects of calcitriol on the expression of Th2 promoters or Th17 promoters in DCs

**FIGURE 4:**
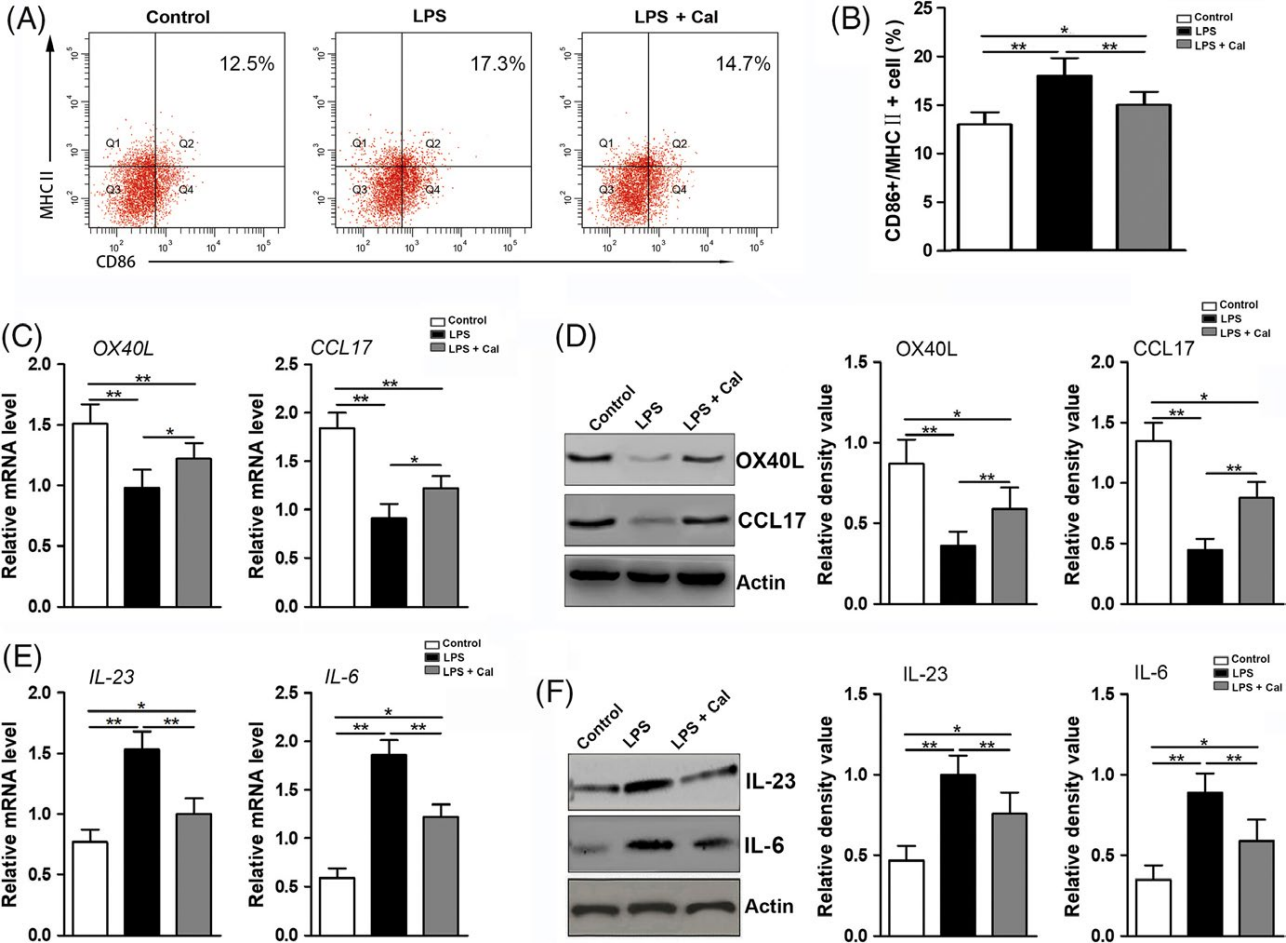
Calcitriol administration modulated the antigen‐presenting function and Th2 or Th17 promoter expression in DCs. A, Representative flow cytometry plots of CD86^+^/MHCⅡ^+^ DCs following incubations in various conditions (control group, LPS group and LPS + Cal group). B, Quantification of the proportion of CD86^+^/MHCⅡ^+^ DCs (assessed by flow cytometry). C, mRNA levels of Th2 promoters (such as *OX‐40L* and *CCL17*) in DCs following incubation in various conditions (control group, LPS group and LPS + Cal group). D, Protein levels of Th2 promoters (such as OX‐40L and CCL17) in DCs (determined by Western blotting) following incubations in various conditions (control group, LPS group and LPS + Cal group; left panel) and semi‐quantitative analysis of the protein expression levels (normalized to the level of actin) in terms of the relative grey density (right panel). E, mRNA levels of Th17 promoters (such as *IL‐23* and *IL‐6*) in DCs following incubations in various conditions (control group, LPS group and LPS + Cal group). F, Protein levels of Th17 promoters (such as IL‐23 and IL‐6) in DCs (determined by Western blotting) following incubations in various conditions (control group, LPS group and LPS + Cal group; left panel) and semi‐quantitative analysis of the protein expression levels (normalized to the level of actin) in terms of the relative grey density (right panel). The data are shown as the mean ± SD; *P < .05 and **P < .01 represent significant differences between the indicated columns

To clarify how calcitriol affects Th cell polarization in an inflammatory environment, the Th cell polarization‐related antigen‐presenting function of DCs was analysed in the present study. Flow cytometry analysis showed that LPS stimulation increased the proportion of CD86^+^/MHCⅡ^+^ DCs, whereas calcitriol intervention inhibited the increase in the proportion of CD86^+^/MHCⅡ^+^ DCs (Figure 4A,B). In terms of qRT‐PCR and Western blot analyses, calcitriol increased the expression levels of Th2 promoters (such as OX‐40L and CCL17) in DCs (Figure 4C,D) but decreased the levels of Th17 promoters (such as IL‐23 and IL‐6) in DCs (Figure 4E,F).

## 4 DISCUSSION

Previously, we found that calcitriol could not only suppress LPS‐induced bone loss but also regulate Th cell polarization in experimental periodontitis.^14^ However, whether the immunomodulatory effects of calcitriol on Th cells were associated with the activation of osteoclasts remained unexplored. Previous studies showed that Th17 cells are crucial resources of proinflammatory products, such as RANKL and IL‐17, which could induce the resorption of bone,^37^ and the tissue destruction of periodontitis is related to the decreased protective effect of the Th2 cell and its cytokine (IL‐4).^4^ Therefore, decreasing Th17 polarization and reinforcing Th2 polarization could represent a significant method for alleviating bone destruction. LPS produced by periodontal pathogens performs essential roles in the destruction of periodontal tissues by affecting host acquired immune responses, particularly by regulating and activating antigen‐presenting cells such as DCs. These activated DCs are capable of inducing Th cells to undergo proinflammatory polarization (Th1 and Th17 phenotypes), release proinflammatory cytokines and destructive proteins to limit pathogen invasion.^38^ Given that a large number of cells (DCs and CD4+ Th cells) are required, primary cells in the present study were obtained from spleen tissue. Accumulated evidence has indicated that DCs and CD4+ T cells from spleen can be used to detect the role of the host immune response on periodontitis and DCs from mouse spleen could exert an antigen‐presenting function to induce CD4+ T responses as well.^39^, ^40^ After Th cells and DCs were successfully obtained, the effects of calcitriol administration on Th cell polarization were assessed in the inflammatory environment with a transwell system. Directly coculturing the DCs and CD4+ T cells increases the difficulty of separating CD4+ T cells from DCs. Moreover, our CFSE‐based flow cytometry assay showed that DCs could perform similar antigen‐presenting functions in a direct co‐incubation system and indirect transwell co‐incubation system (Figure 1A,B, Figure S2A,B). Flow cytometry analysis showed that calcitriol intervention intensified Th2 polarization and inhibited Th17 polarization in the inflammatory environment, which indicates that calcitriol intervention can weaken the inflammatory response by regulating Th cell polarization (Figure 1C,D). Consistently, our in vivo evidence has demonstrated that calcitriol plays an anti‐inflammatory role by decreasing the infiltration of inflammatory cells in LPS‐induced periodontitis.^14^ Other related studies have also confirmed that calcitriol treatment attenuates LPS‐induced bone loss by enhancing Th2 polarization and inhibiting Th17 polarization at the transcriptional level.^41^, ^42^ In addition, qRT‐PCR and Western blotting were used to detect the expression of Th2/Th17 polarization‐related markers, such as GATA3, STAT5 and IL‐4 for Th2 polarization and RORγT, STAT3 and IL‐17 for Th17 polarization. In accordance with the aforementioned flow cytometry‐based analysis, we found that calcitriol intervention elevated the mRNA and protein levels of Th2‐specific transcription factors (GATA3 and STAT5) but reduced the mRNA and protein levels of Th17‐specific transcription factors (RORγT and STAT3) (Figure 1E‐H). Among these markers, GATA3 and STAT5 play essential roles in generating IL‐4 during the process of Th2 polarization, and the production of IL‐4 can enhance Th2 polarization and augment GATA3 expression in autoimmune diseases.^27^, ^28^, ^30^ High GATA3 expression levels have been shown to be closely related to the protective effect of Th2 cells in the recovery phase of non‐surgical periodontal treatment.^11^ However, the crosstalk between STAT5 and calcitriol in immune responses still has not been evaluated in systematic studies, and discussing the regulatory effect of calcitriol on STAT5 in the immune response may be of significance in the treatment of immune diseases. Regarding Th17 polarization‐related markers, increased expression of STAT3 and RORγT has been found to be closely linked to alveolar bone loss induced by high IL‐17 expression in mice with periodontitis.^16^, ^29^, ^30^ Moreover, calcitriol treatment has also been reported to inhibit cytokine production in Th17 cells at the post‐transcriptional level by impeding the activation of STAT3 and RORγT and boosting endoplasmic reticulum stress in vitro.^31^

Given that RANKL produced by macrophages and lymphocytes can facilitate osteoclast differentiation and maturation, leading to RANKL‐induced bone resorption, we selected RANKL expression in Th cells as a parameter to assess osteoclastogenesis in the present study. The effect of calcitriol intervention on RANKL expression was analysed by qRT‐PCR and Western blot analyses. The results showed that calcitriol intervention could downregulate the RANKL expression level in Th cells (Figure 2A,B), which matches our previous study showing that calcitriol administration inhibits RANKL expression in gingival tissues in a rat periodontitis model.^14^ Th17 cells, as an essential source of RANKL, are able to facilitate mature osteoclast functions and induce rapid conversion from a bone‐non‐resorptive environment to a bone‐resorptive environment during the process of inflammatory bone destruction.^43^ Thus, IL‐17^+^/RANKL^+^ cells were detected by flow cytometry and immunofluorescence assays, and the results demonstrated that calcitriol intervention exerted a suppressive effect on the proportion of IL‐17^+^/RANKL^+^ cells (Figure 2C‐F). Indeed, recent studies have demonstrated that calcitriol potently inhibits RANKL expression by suppressing RORγT and IL‐17 expression in Th17 cells^16^ and that this inhibitory effect can debilitate osteoclastogenesis through JAK‐2/STAT‐3 and p38 MAPK/NF‐κB signalling in rheumatoid arthritis.^44^ However, the role of RANKL expression in Th17‐induced bone loss during periodontitis warrants further investigation.

To further validate that calcitriol influences osteoclastogenesis via Th cell polarization, we used TRAP staining, qRT‐PCR and Western blot analyses to evaluate the effect of calcitriol‐treated Th cells on RAW264.7 cell osteoclastogenesis. In comparison to incubation without calcitriol, incubation with calcitriol led to fewer RAW264.7 cells exhibiting positive TRAP staining and RAW264.7 cells with lower levels of NFATc1 and MMP‐9 (Figure 3A‐D), which suggests that Th cell polarization is a crucial part of the process of the calcitriol‐mediated suppression of osteoclastogenesis. NFATc1 and MMP‐9 were selected as osteoclastogenesis‐related markers in this research.^33^ NFATc1, as an essential transcription factor in osteoclasts, can regulate cathepsin K and TRAP during bone resorption and be inhibited by low levels of RANKL due to low Th17 and high Th2 responses.^8^, ^45^ In LPS‐induced bone destruction, thymol could inhibit bone loss by the activation of ERK, JNK and AKT and downregulation of NFATc1, C‐FOS, MMP‐9 and cathepsin K.^46^ MMP‐9, as a crucial product from RAW264.7 cells, can accelerate collagen degradation by inhibiting the protective effects of antitrypsin and activating MMP‐13.^47^ Therefore, calcitriol intervention could be a useful measure to attenuate bone resorption in periodontitis by reducing NFATc1 and MMP‐9.

As an immunological adjuvant, calcitriol has been found to directly downregulate the expression of Th1/Th17‐related cytokines (such as IFN‐γ and IL‐17) and upregulate the expression of Th2/Treg‐related cytokines (such as IL‐4 and IL‐10) in vivo.^48^, ^49^ However, not only Th cells but also antigen‐presenting cells, especially DCs, have been implicated in the processes of host acquired immune responses.^38^ Therefore, the influence of the antigen‐presenting function of DCs on Th cell polarization in response to calcitriol administration was assessed in the present study. Previous studies have demonstrated that LPS stimulation can upregulate the expression of MHCⅡ and CD86 in DCs,^50^ whereas several immunoregulatory agents, such as conjugated linoleic acid and curcuma kwangsiensis, can reduce the expression of MHCⅡ and CD86 in DCs and suppress Th1/Th17 polarization under inflammatory stimulation.^51^, ^52^ By combining the transcription factor NF‐κB and a regulatory region, LPS treatment can enhance the antigen‐presenting function of DCs and subsequently lead to a high level of proinflammatory Th polarization and a low level of anti‐inflammatory Th polarization.^26^, ^53^ Similarly, in our study, calcitriol treatment increased the expression of MHCⅡ and CD86 on the surface of DCs and restrained the ability of DCs to stimulate the proliferation of mixed allogeneic lymphocytes (Figure 4A,B).

It has been reported that a high level of OX‐40L in DCs can enhance Th2 polarization in vivo, which is considered an essential protective mechanism against tissue destruction in some disorders such as infectious, inflammatory and autoimmune diseases.^54^ Additionally, both OX‐40L and CCL17 in DCs can trigger Th2 polarization, which induces a low inflammatory response and plays a protective role in allergy under the effect of thymic stromal lymphopoietin.^35^ Our data indicate that calcitriol has the crucial potential to block inflammatory reactions by upregulating the DC‐OX‐40L/CCL17‐Th2 axis (Figure 4C,D). However, there is still a lack of related reports regarding the effect of the DC‐OX‐40L/CCL17‐Th2 axis on inflammation‐related bone resorption. As proinflammatory cytokines are mainly secreted by DCs, IL‐23 and IL‐6 can regulate Th17 polarization and contribute to bone loss induced by innate immunity.^25^, ^36^, ^55^ Moreover, IL‐23 receptor signalling is capable of activating JAK‐STAT, NFκB and RORγT, which induce osteoclastogenesis through Th17 cells and their product (IL‐17).^55^, ^56^ Interestingly, *Porphyromonas gingivalis* inducing high amounts of IL‐23 and IL‐6 can initiate Th17 cell‐dependent adaptive immunity.^57^ The current study showed a similar result: LPS stimulation promoted the expression of IL‐6 and IL‐23 in DCs (Figure 4E,F). However, more importantly, calcitriol intervention was found to downregulate the IL‐6 and IL‐23 expression levels (Figure 4E,F). To attenuate tissue destruction, several reports have investigated valid ways to inhibit the inflammatory response by modulating the IL‐23/IL‐17/Th17 axis of the immune system.^55^, ^58^ Therefore, it is particularly important to define the mechanism of how calcitriol affects antigen presentation by DCs.

In conclusion, the results reported in the current study support the conclusion that calcitriol can suppress inflammation‐induced osteoclastogenesis in vitro by changing the proportion and function of Th cell subsets, which indicates that calcitriol may be a promising therapeutic agent for the treatment of chronic periodontitis.

## CONFLICT OF INTEREST

The authors declare that they have no competing interests.

## AUTHOR CONTRIBUTIONS

C.‐S. B., X. L., Y.‐L. H. and F.‐M. C.: conception and design; C.‐S. B., X. L., H.‐L. Q., L.‐J. S. and B.‐M. T.: collection and assembly of data; C.‐S. B., X. L., B.‐M. T. and Y. A.: data analysis and interpretation; C.‐S. B., X. L., B.‐M. Tian, Y.‐L. H. and F.‐M. C.: manuscript preparation; and B.‐M. T. and F.‐M. C.: financial support.

## Supporting information

Fig S1

Fig S2

## Data Availability

All data generated or analysed during this study are included in this article.
