# Supplementary figures and images for: Calcitriol inhibits osteoclastogenesis in an inflammatory environment by changing the proportion and function of T helper cell subsets (Th2/Th17)
Source: Cell Prolif. 2020 May 13;53(6):e12827. doi: 10.1111/cpr.12827 (PMC7309596; doi:10.1111/cpr.12827)

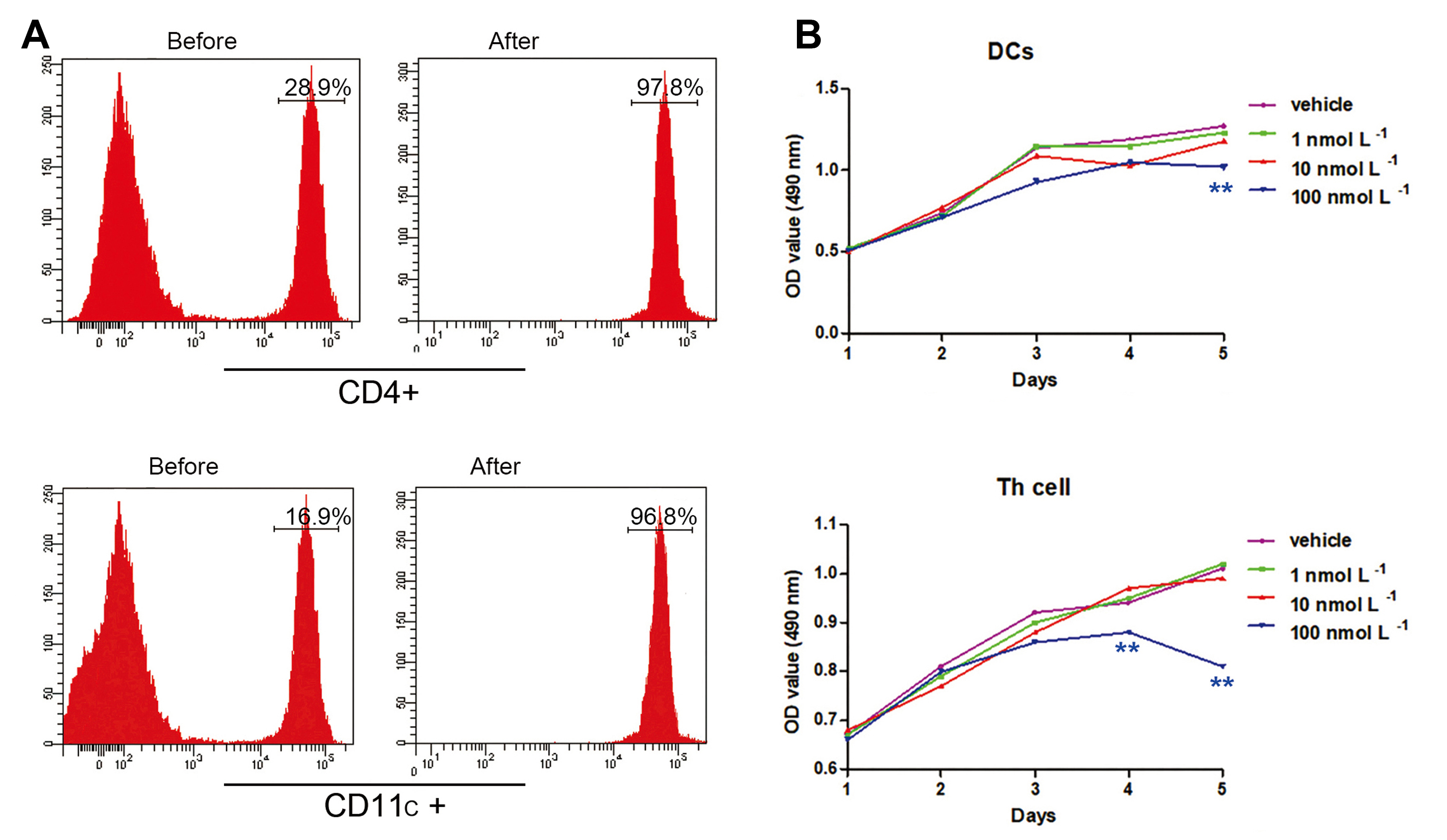

Supplement: Supplementary file 1 — Fig S1 [file CPR-53-e12827-s001.tif]

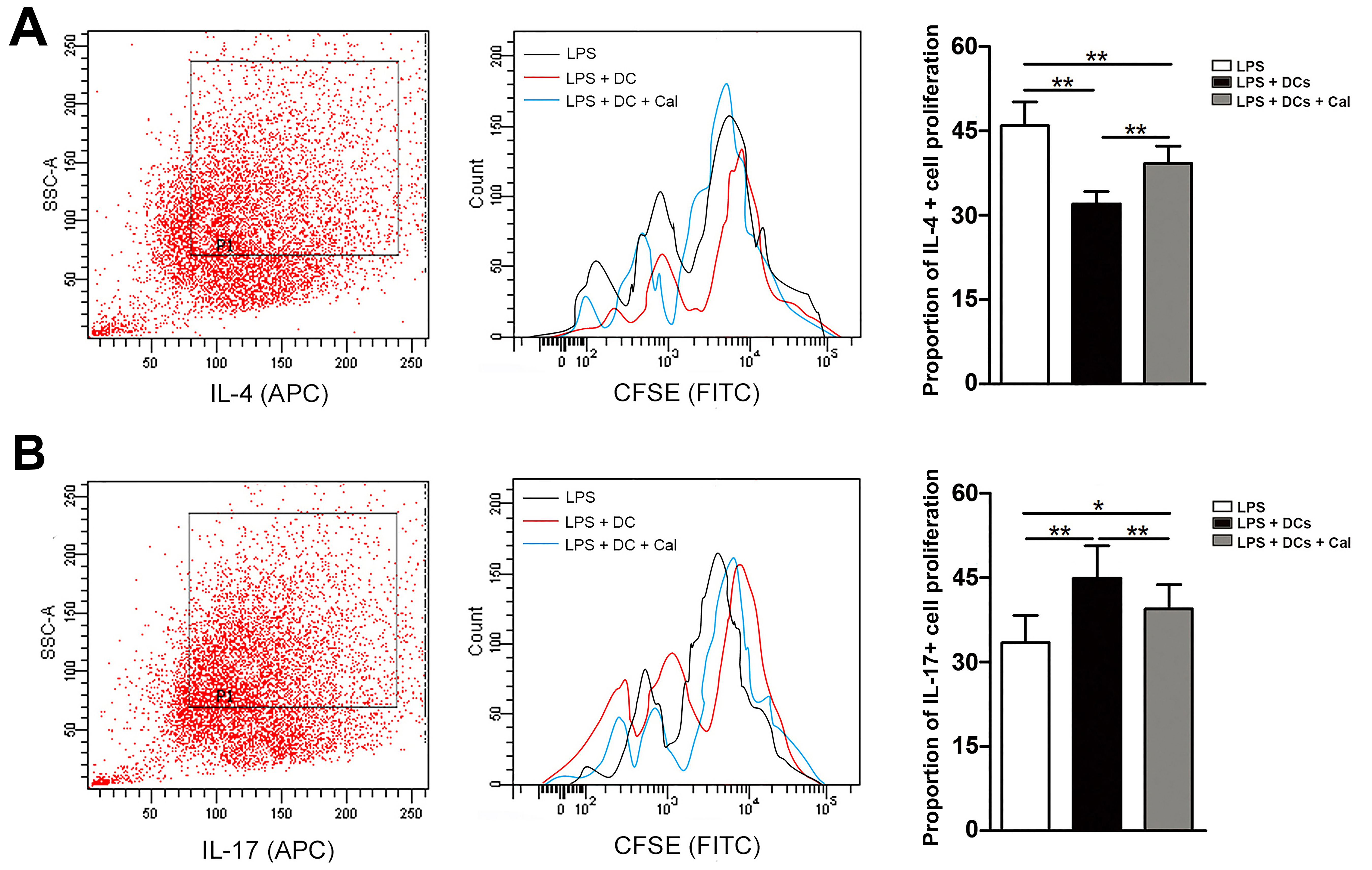

Supplement: Supplementary file 2 — Fig S2 [file CPR-53-e12827-s002.tif]
